# Unraveling a Rare Case of Pediatric Solid Pseudopapillary Neoplasm With a Replaced Common Hepatic Artery Arising From the Superior Mesenteric Artery

**DOI:** 10.7759/cureus.73332

**Published:** 2024-11-09

**Authors:** Niyas Ahamed, Padmanabhan S, Karthikeyan Srinivasan, Venkatesan P, Sastha Ahanatha Pillai

**Affiliations:** 1 Surgical Gastroenterology, Madurai Medical College, Madurai, IND; 2 General Surgery, Sri Ramachandra Institute of Higher Education and Research, Chennai, IND

**Keywords:** hepatic artery anomaly, pediatric tumor, replaced common hepatic artery, solid pseudopapillary neoplasm, whipple’s pancreaticoduodenectomy

## Abstract

Pancreatic solid pseudopapillary epithelial neoplasm (SPEN) is a rare pancreatic tumor with low-grade malignant potential. They often present in young women in their second and third decade of life, with only a small minority concerning children. It has a good prognosis, with a five-year survival rate of up to 97%. A common hepatic artery (CHA) arising from the superior mesenteric artery (SMA) is a rare occurrence. A 12-year-old girl, admitted with features of obstructive jaundice, was evaluated to have a heterogeneously enhancing mass lesion of size 7.5 × 7.2 cm involving the head of the pancreas on contrast-enhanced CT of the abdomen (CECT). CT angiogram showed a CHA trunk arising from the SMA and a type V hepatic arterial variation. Whipple’s pancreaticoduodenectomy was done for the patient, and the postoperative period was uneventful. The subsequent histopathology report was confirmatory for SPEN, with an R0 margin of resection. SPENs of the pancreas are extremely rare, and after surgical resection, they often have an excellent long-term prognosis. Variations in hepatic artery anatomy, as in this case, need meticulous dissection to avoid inadvertent vascular insult.

## Introduction

Pancreatic solid pseudopapillary epithelial neoplasm (SPEN or Frantz’s tumor) is a rare tumor, accounting for 1-2% of all pancreatic malignancies [1-4]. Though considered benign historically, SPENs have now been redefined as low-grade malignant tumors with a low incidence of metastases and high survival rates. Despite being classified as low-grade malignant tumors, up to 10-15% of cases are reportedly aggressive. Most patients are females in their second and third decade of life, with children constituting only a small proportion among them [1,5]. Although they are often diagnosed incidentally, they can present as a slow-growing abdominal mass with abdominal pain. However, despite their malignant potential, curative R0 resections have been associated with an excellent long-term prognosis and a five-year survival rate of up to 97% [6-8]. A replaced common hepatic artery (CHA) arising from the superior mesenteric artery (SMA) is a rare phenomenon whose incidence is between 1.5% and 4.0% [9].

## Case presentation

A 12-year-old girl was admitted with complaints of jaundice, clay-colored stools, and pruritus for one week. She also reported right upper quadrant abdomen pain and backache for three days. During her illness, she noticed a lump in the right upper quadrant of her abdomen. On clinical examination, she had a firm to hard mass, measuring 6 × 5 cm, occupying the epigastric and right hypochondriac region. An enlarged and distended gallbladder extending to the right iliac fossa was also palpable. 

**Figure 1:**
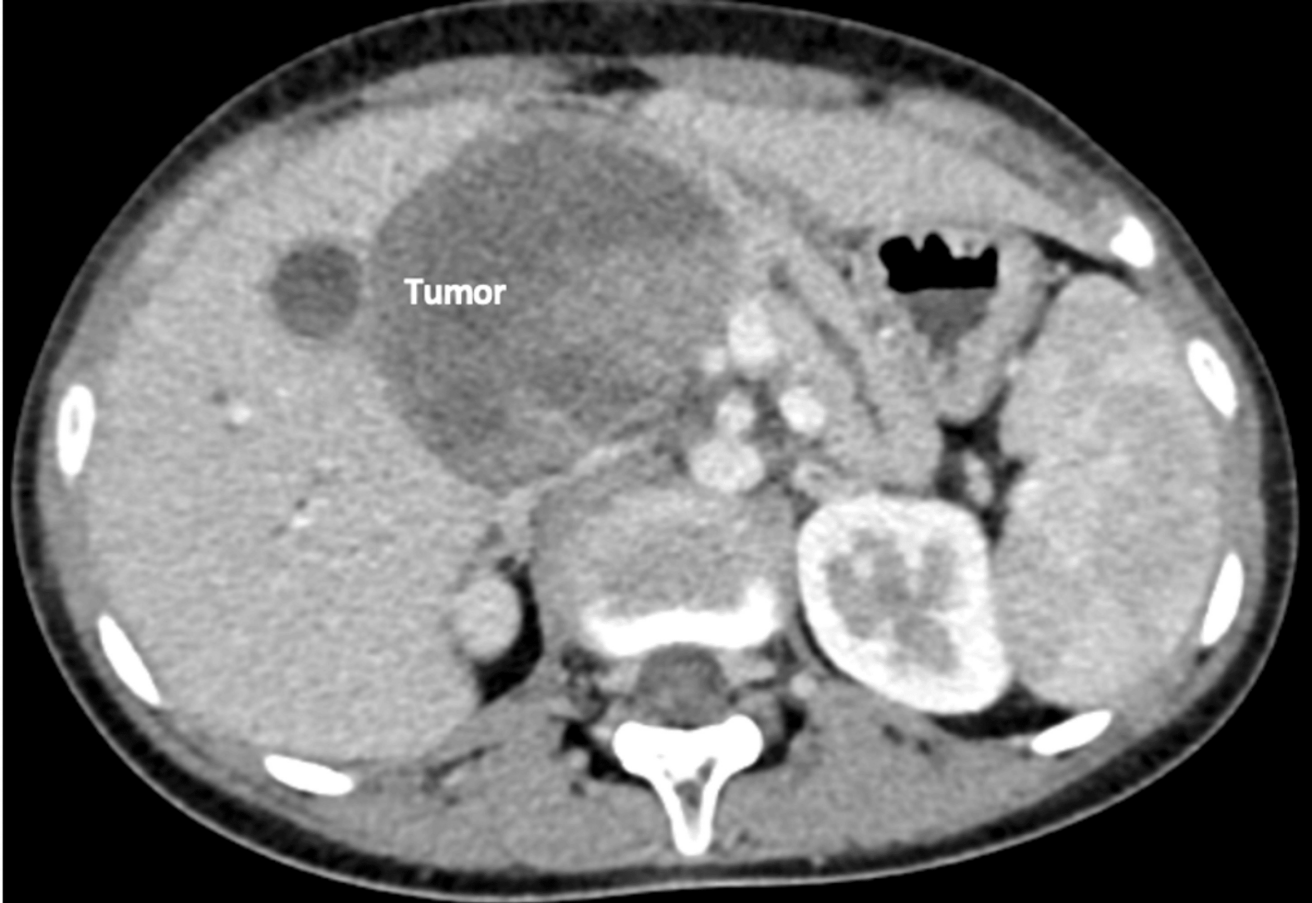
CECT abdomen (axial cut) The tumor labeled is seen to have both solid and cystic areas within it, a pathognomonic feature of SPEN SPEN, solid pseudopapillary epithelial neoplasm; CECT, contrast-enhanced CT of the abdomen

**Figure 2:**
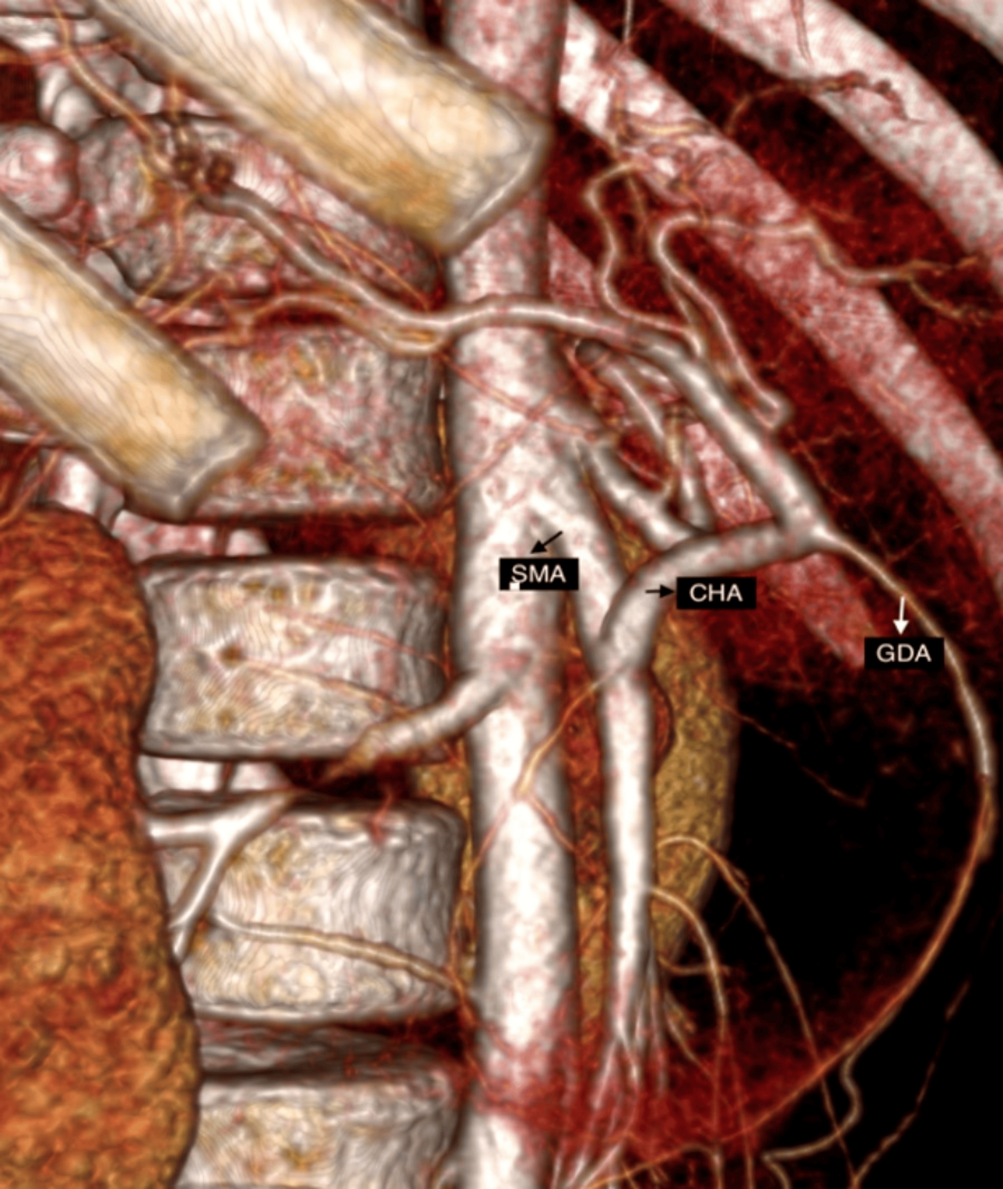
Replaced CHA seen arising from SMA (sagittal section) GDA, gastroduodenal artery; SMA, superior mesenteric artery; CHA, common hepatic artery

**Figure 3:**
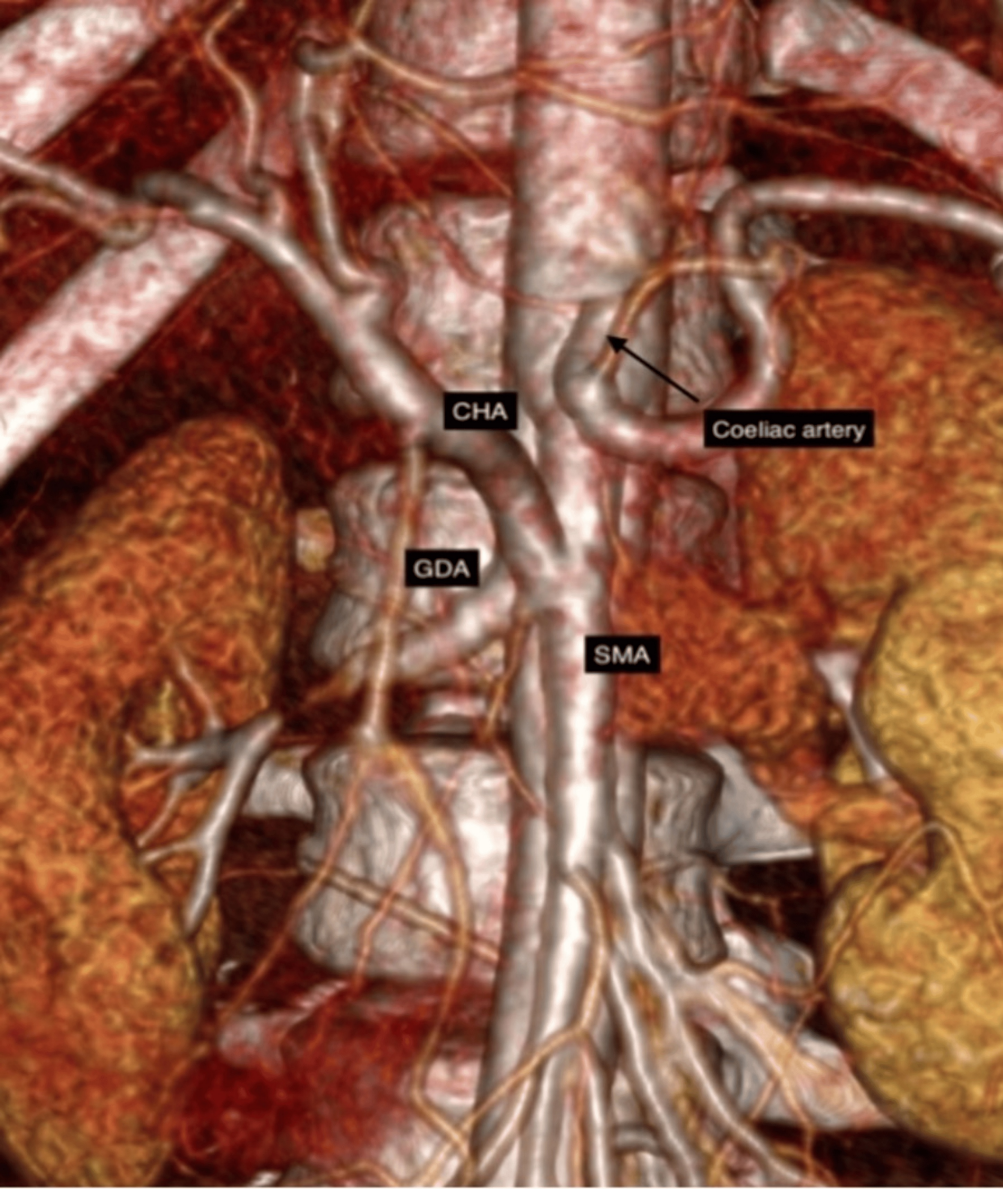
Replaced CHA seen arising from SMA (coronal section) GDA, gastroduodenal artery; SMA, superior mesenteric artery; CHA, common hepatic artery

Contrast-enhanced CT of the abdomen (CECT) revealed a heterogeneously enhancing mass lesion of size 7.5 × 7.2 cm involving the head of the pancreas, with areas of necrosis, hemorrhage, and faint calcification without distant metastases (Figure 1). The portal vein/superior mesenteric vein (SMV) was displaced medially. CT angiogram demonstrated a CHA trunk arising from the SMA (Figures 2, 3) and a type V (Hiatt et al.) hepatic arterial variation (Table 1) [10].

**Table 1:** Hiatt’s classification of hepatic arterial anatomy variations SMA, superior mesenteric artery; CHA, common hepatic artery; RHA, right hepatic artery; LHA, left hepatic artery

Type	Description
1	Normal
2	Replaced or accessory LHA
3	Replaced or accessory RHA
4	Replaced or accessory RHA and replaced or accessory LHA
5	CHA from SMA
6	CHA from aorta

The common bile duct measured 1.3 cm, with intra-hepatic biliary radical dilatation, and the main pancreatic duct (MPD) was non-dilated (2 mm). Total bilirubin was 3.71 mg/dL, carcinoembryonic antigen (CEA) was 2.1 ng/mL (normal, <5 ng/mL), and carbohydrate antigen (CA) 19-9 was 30.2 U/mL (normal, <35 U/mL). Upper GI endoscopy showed an extrinsic compression onto the stomach lumen. The preoperative working diagnosis was that of SPEN. 

**Figure 4:**
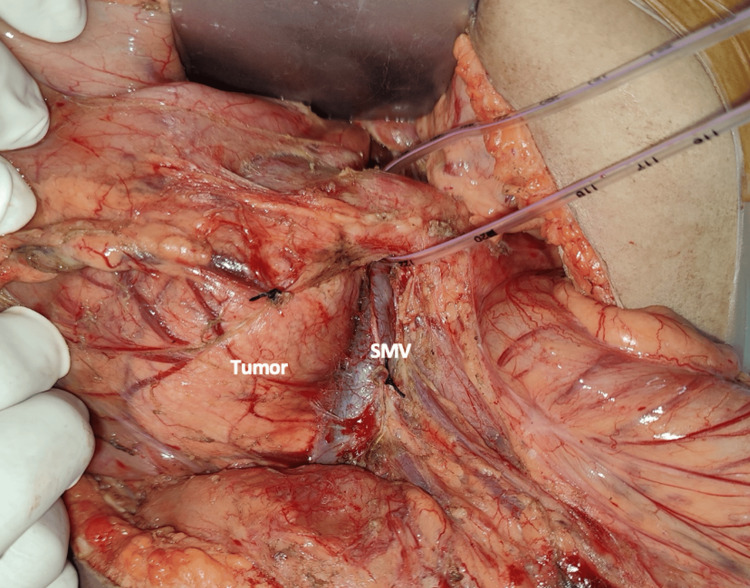
Tumor is seen in close proximity to mesenteric vessels The close proximity of the mesenteric vessels to the tumor mandated a meticulous dissection. SMV, superior mesenteric vein

**Figure 5:**
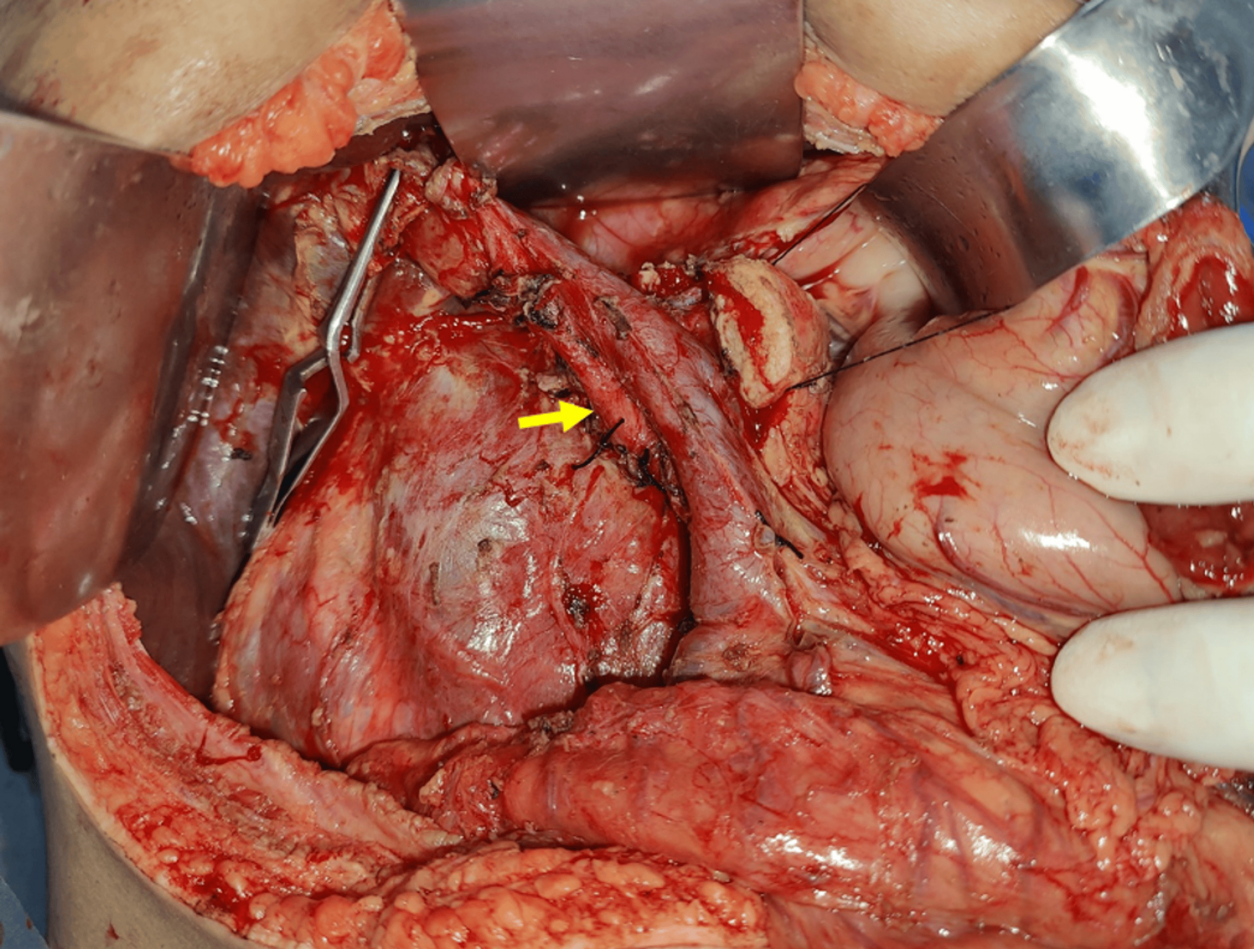
Replaced CHA arising from SMA (yellow arrow) SMA, superior mesenteric artery; CHA, common hepatic artery

The patient was taken up for Whipple’s pancreaticoduodenectomy surgery. Intraoperative findings confirmed the mass’s close adherence to the nearby vascular structures (SMA/portal vein) (Figure 4). The presence of a CHA trunk arising directly from the SMA was confirmed intraoperatively (Figure 5). The CHA, originating from the SMA, ran posterior to the pancreas and the portal vein (type IV-P; Higashi and Hirai) [11]. Reconstruction included pancreatico-jejunostomy, hepatico-jejunostomy, and gastro-jejunostomy, with a feeding jejunostomy. The surgery was completed with <50 mL of blood loss. Postoperatively, there was no pancreatic leak. The patient was started on oral intake from the postoperative day (POD) 4, drain tubes were removed on POD 7, and the patient was discharged on POD 12. The postoperative period was uneventful. The postoperative histopathology report confirmed SPEN with an R0 margin of resection.

## Discussion

SPENs of the pancreas are rare, solitary, large, and well‑circumscribed solid tumors, which most often involve the pancreatic tail. They are bound by a thick fibrous wall within which areas of cystic degeneration are present [12]. SPENs constitute around 1-2% of all exocrine pancreatic neoplasms, but in children, SPENs comprise between 6% and 17% of all pancreatic tumors [13]. The median age at presentation in India is 24 years, with a male-to-female ratio of 1:6. The most common clinical presentation is abdominal pain [14]. The risk of malignancy is relatively low, around 10-20%.

SPENs originate from the genital ridges that contain pluripotent cells that adhere to the pancreas during early embryogenesis [13]. They exhibit foci of necrosis, hemorrhage, and cystic changes, producing a variegated appearance. The variable appearance of SPENs has been attributed to vascular ischemia [15]. The presence of pseudo-rosettes and pseudopapillary patterns is pathognomonic of SPENs. The presence of cercariform cells distinguishes it from neuroendocrine pancreatic tumors [13]. In immunohistochemistry, these tumors stain positively for ß-catenin, CD99, vimentin, neuron-specific enolase (NSE), alpha-1-antitrypsin, and alpha-1-antichymotrypsin. Tumor markers like CEA or CA19-9 are seldom elevated.

Characteristically, on CECT, SPENs enhance similar to the surrounding pancreatic parenchyma and appear as large, well‑delineated, and encapsulated masses that contain peripheral solid and central cystic components without dilatation of the MPD. En bloc (R0) surgical resection remains the treatment of choice. Following an R0 resection, recurrence is uncommon, even without adjuvant therapy [16]. The role of neoadjuvant and adjuvant therapy is very limited, highlighting the need for meticulous surgery [14]. 

Variations in hepatic artery anatomy are not uncommon. The celiac trunk normally trifurcates into the left gastric artery (LGA), splenic artery, and CHA, the incidence of which is 75%. The CHA, after branching into the gastroduodenal artery (GDA) and right gastric artery, continues as the proper hepatic artery. The latter gives rise to the right hepatic artery (RHA) and the left hepatic artery (LHA). 

During pancreaticoduodenectomy, an unrecognized anatomical variation of liver and pancreatic arteries can lead to catastrophic vascular injuries, resulting in intraoperative bleeding and/or postoperative liver failure or bowel ischemia [17]. Our patient had a rare (type V) hepatic arterial variation as described by Hiatt et al. [10], wherein a replaced CHA was seen arising from the SMA, whose incidence is between 1.5% and 4.0% [9]. Higashi and Hirai further classified hepatic arterial variations arising from the SMA into four types with subgroups, to which our patient belonged to type IV-P [11].

The importance of sparing the replaced CHA during pancreaticoduodenectomy lies not only in preventing hepatic ischemia but also in avoiding a breakdown of biliary-enteric anastomosis. This is because the blood supply to the proximal bile duct after pancreaticoduodenectomy is entirely dependent on the RHA [18]. In such cases, it is prudent to start with the pancreatic parenchymal division before completing CHA and GDA dissection, as the CHA can be mistaken for the GDA. The sheer size of the tumor, along with the altered arterial anatomy, presented a technical challenge in our case. Meticulous dissection and skeletonization of the CHA (running dorsal to the head of the pancreas containing the tumor) and the GDA prevented a major vascular insult in this patient.

A keen observation and proper analysis of the preoperative CT scan by the surgeon play a pivotal role not only in defining the resectability of the tumor but also in finding out vascular abnormalities. As in this case, a careful insight into the arterial variation prevented intraoperative turmoil. This paves the way for a better orchestration of the surgery, avoiding inadvertent intraoperative injuries. 

## Conclusions

SPEN is a rare pancreatic tumor in adult females, and a pediatric presentation of this tumor makes it still rare. The patient had a replaced CHA arising from the SMA (type V Hiatt), which is again a rare occurrence. Careful dissection and preoperative anatomical orientation led to a successful outcome for this patient.
